# Putative protective genomic variation in the Lithuanian population

**DOI:** 10.1590/1678-4685-GMB-2023-0030

**Published:** 2024-04-15

**Authors:** Gabrielė Žukauskaitė, Ingrida Domarkienė, Tautvydas Rančelis, Ingrida Kavaliauskienė, Karolis Baronas, Vaidutis Kučinskas, Laima Ambrozaitytė

**Affiliations:** 1 Vilnius University, Faculty of Medicine, Institute of Biomedical Sciences, Department of Human and Medical Genetics, Vilnius, Lithuania.

**Keywords:** Allele frequency analysis, complex diseases, effect variants, genotyping, positive selection, protective alleles

## Abstract

Genomic effect variants associated with survival and protection against complex diseases vary between populations due to microevolutionary processes. The aim of this study was to analyse diversity and distribution of effect variants in a context of potential positive selection. In total, 475 individuals of Lithuanian origin were genotyped using high-throughput scanning and/or sequencing technologies. Allele frequency analysis for the pre-selected effect variants was performed using the catalogue of single nucleotide polymorphisms. Comparison of the pre-selected effect variants with variants in primate species was carried out to ascertain which allele was derived and potentially of protective nature. Recent positive selection analysis was performed to verify this protective effect. Four variants having significantly different frequencies compared to European populations were identified while two other variants reached borderline significance. Effect variant in *SLC30A8* gene may potentially protect against type 2 diabetes. The existing paradox of high rates of type 2 diabetes in the Lithuanian population and the relatively high frequencies of potentially protective genome variants against it indicate a lack of knowledge about the interactions between environmental factors, regulatory regions, and other genome variation. Identification of effect variants is a step towards better understanding of the microevolutionary processes, etiopathogenetic mechanisms, and personalised medicine.

## Introduction

Each population may have some exceptional genetic characteristics which might differentially affect health, lifestyle and evolution through adaptation. The Lithuanian population is genetically close to neighbouring European populations, for example, Slavs and Finno-Ugrians (Kasperavičiūtė et al., 2004). In addition, Y chromosome single nucleotide polymorphism (SNP) haplogroup analysis in the same study showed that Lithuanians are genetically closest to Latvians and Estonians. Recent studies show that Lithuanian population is homogeneous, genetically differentiated from neighbouring populations but within the general expected European context (Urnikyte et al., 2019). The characterization of genome variation in different populations, such as Lithuanian or any other, is important in order to understand the differences between populations and assessing these differences may be useful in understanding the biological mechanisms of adaptation, survival, as well as complex traits and diseases. Specific genomic loci and variants associated with adaptation vary qualitatively and quantitatively between populations and fluctuate over time, in part, due to microevolutionary processes, such as genetic drift and natural selection. In a changing environment, genetic variants that were once advantageous or neutral in relation to certain traits may become deleterious (and vice versa) and lead to changes in the genetic architecture of a population undergoing adaptation (Merilä et al., 2001). 

To understand the mechanisms of complex diseases and traits, the question of natural selection and adaptation through the genomic variation fluctuation process in the population over a period of time has to be answered. A critical point in understanding aforementioned mechanisms is that some derived genomic variants cannot simply be categorised as risk or protective (the neutral variation analysis is off the scope of this study), because of conflicting interpretations of their effect. Thus, we refer to these variants as effect variants as they can have various consequences such as the prevalence of complex diseases leading to high mortality: hypertension (Hancock et al., 2008), coagulation changes (Dahlbäck, 2008), and hyperlipidaemia (Stengård et al., 1995). Such research findings have implications for population-specific (geographically and ethnically) diagnosis worldwide (Butler et al., 2017) and defining frequency of effect variants, origin and impact to protein structure is a necessity.

Effect variants which provide selective advantage against certain diseases are usually rare between individuals who have a disease, and tend to become common in part of the population that does not have it (Butler et al., 2017). That is why our analysis includes not only rare but also common effect variants. Besides, most of these variants are likely to be common in biologically redundant genes, thereby escaping the effects of purifying selection and preserving these variants at high frequencies in various populations (MacArthur et al., 2012). Theoretically, it can be simplified as follows: if a person has an effect variant that protects against certain disease (e.g. HIV) and environment provides necessary selective pressure, this person may less likely have the disease and more likely to pass this variation to one’s offspring due to positive selection. The beneficial allele at the selected locus increases in frequency while linked neutral variation diminishes, creating a so-called selective sweep. Based on this logic, complex disease rates in the population should drop in the future. However, complex disease rates are steady and one of the reasons might be exploding growth of the human population, which results in an accumulation of extremely rare variants (Maher et al., 2012). Another reason is reduction of intensity of purifying selection and more frequent fixation of nonsynonymous mutations while getting older (Cheng and Kirkpatrick, 2021). Genome-wide association studies (GWASs) under-represent low frequency variants (minor allele frequency [MAF] 0.5-2%) and rare variants (MAF<0.5%) that could underlie much of the unexplained heritability of many complex traits (Lee et al., 2014). In addition, minor alleles are more likely to be characterised as risk alleles in published GWASs on complex diseases because minor alleles are more easily detected as risk alleles in GWASs (Kido et al., 2018).

The origin of the effect allele must also be addressed. Every disease-associated single nucleotide variant (SNV) consists of two alleles. When the specific environmental context and selective pressures acting on a given population are unknown, a common practice to ascertain whether a nonsynonymous SNV is protective (i.e., the derived allele is protective) is to deduce which allele is derived and which is ancestral. Ancestral alleles tend to have neutral effect (Butler et al., 2017). Therefore, the protective nature of genomic variants can be considered when the allele is derived. However, it is important to take into account that certain ancestral alleles may provide adaptive advantages in new environments, leading to their selection and maintenance in specific populations out of ancestral populations.

Frequency and origin are not the only criteria for including effect variants in this study. Mostly non-synonymous single nucleotide effect variants were chosen for this study to analyse those that affect the structure of the protein and may have a function-altering effect. Many effect variants protect against disease by disrupting protein function, typically via loss-of-function or gene knockout effects, and have an impact on clinically relevant phenotypic effects. In this case, most of the functionally relevant loss-of-function variants should be removed by purifying selection (Harper et al., 2015). However, recent studies have shown that synonymous variants can also influence the amount of protein that is produced; so-called optimal codons are faster for cells to process and lead to increased protein production (Dhindsa et al., 2020). This reveals that synonymous variants likely play an underestimated role in human genomic variation. That is the reason why we included some of the synonymous effect variants in our analysis as well.

In this study, we aimed not only to characterize effect (risk, or protective) variants in the genomes of the individuals from the Lithuanian population, but also to evaluate the possible influence of positive natural selection on genomic loci in which these variants are. If genomic loci of the effect variants are under positive selection, it may be due to the advantageous nature of the genomic loci and the effect variant itself. Therefore, identification of signals of recent positive selection provides information about the adaptation of modern humans to local conditions. For example, in the Urnikyte et al. (2019) study, among the top signatures of positive selection detected in Lithuanians, there were several candidate genes identified which were related to diet (*PNLIP*, *PPARD*), pigmentation (*SLC24A5*, *TYRP1*, *PPARD*), and the immune response (*BRD2*, *HLA-DOA*, *IL26* and *IL22*). This shows that the positive selection directly affects the lifestyle and certain traits (i.e. pigmentation), related to adaptation in the local population. In the same Urnikyte *et al*. (2019) study, candidate loci affected by positive selection were identified using traditional (F_ST_ and XP-EHH) analysis methods. To complement previous results with novel, unique, and more detailed results, this study aimed to analyse the influence of positive selection on a set of particular effect variants (and genomic loci) using RAiSD tool (Alachiotis and Pavlos, 2018) created to detect positive selection signatures.

The identification of effect variants, a better understanding of their role in microevolutionary processes, and interactions between these variants could provide the possibility to characterise candidate genomic regions and specify their role across different populations (Chattopadhyay and Lu, 2019; Li et al., 2020). Moreover, we provide a discussion on challenges of effect variant role assessment in the context of complex diseases, traits, and potential positive selection in a local population, and our results might contribute to the quickly evolving opportunities of personalised medicine.

## Subjects and Methods

### Study design

First, the aim was to make a catalogue (list) of effect variants (144 variants were selected according to the scientific literature and databases). Later, evaluation of variant frequency in the Lithuanian population was performed, and compared with other European populations. As this study dataset does not cover whole genomes, i.e. for some individuals, whole exome, and for some, microarray data was obtained, not all genomic positions were covered. Therefore, not all variants on the list could be evaluated in this dataset. 

Second, positive selection analysis was chosen to justify the possible protective effect if a variant falls within a region under positive selection. The dataset in this study was primarily used in the study of Urnikyte et al. (2019) and was re-evaluated using a new positive selection analysis method. Additionally, the main aim here was to conduct a targeted analysis for the specific list of variants and discuss their potential protectiveness in the Lithuanian population, which had not been performed previously. Selective sweep detection in this study was used as a tool to analyse genomic loci in which the statistically significant variants were present.

### Participants and samples

The study was conducted according to the ethical standards and was approved by the Vilnius Regional Research Ethics Committee (approval No. 158200-05-329-79 and No. 2019/4˗1119˗612). Informed consent was obtained from all individuals involved in the study. The study group included 475 unrelated, self-reported healthy individuals (239 women and 236 men) of Lithuanian descent (with at least three generations living in Lithuania).

DNA was extracted from peripheral blood leukocytes using the phenol-chloroform-isoamyl alcohol method according to laboratory-approved methodology or using an automated TECAN Freedom EVO^®^200 system (Tecan Group Ltd., Switzerland) using Promega beads assay according to the manufacturer’s user guidelines. Concentration and purity of the DNA were determined with a NanoDrop^®^ spectrophotometer (Thermo Fisher Scientific, Wilmington, DE, USA).

### Catalogue of effect variants

A catalogue of 144 effect variants from the ClinVar (Landrum et al., 2018) and OMIM (https://omim.org/) databases as well as scientific publications (Harper et al., 2015; Butler et al., 2017) was compiled. The criteria for including a variant from the databases were 1) clinical significance review status (protective or uncertain) and 2) count of submissions (more than 1). The criteria for including a variant from scientific publications were 1) the influence of the variant on gene function (i.e., the variant was expected to alter gene function; mostly loss-of-function) and 2) the frequency of the variant (i.e., rare or previously rare alleles, which increased in frequency possibly because of advantageous effect on the phenotype). The catalogue (Table S1) was used to filter out the genotypes of effect variants in the sample group and perform targeted effect variant frequency analyses.

### Genotyping data and statistical analysis

The genotypes were extracted from data of exome sequencing and genome-wide genotyping arrays. *Homo sapiens* genome assembly GRCh37 (hg19) from Genome Reference Consortium was used. Whole exome sequencing was performed using 5500 series SOLiD™ systems protocol guides for 98 individuals of Lithuanian descent. High-throughput genotyping (Illumina HiScanSQ System, Illumina Inc., San Diego, CA, USA) was performed using Illumina Infinium® HD and HTS assay protocol guides (bead chip arrays Illumina 770 HumanOmniExpress-12 v1.0, v.1.1. and Infinium OmniExpress-24v1-2) for 475 individuals of Lithuanian descent. The dataset was re-examined for duplicates. The relatedness of individuals for this dataset was evaluated in the previous study by Urnikyte et al. (2019). 

Quality control of exome sequencing data was performed using LifeScope™ Genomic Analysis Software v2.5. Sequence coverage value of more than 10-fold was considered acceptable (mean quality score of the reference allele: 28 [± 2.3], mean quality score of the new allele: 28.4 [± 1.8]) (Casals et al., 2013).

Genotyping data was quality-controlled and prepared for further analysis by using GenomeStudio v2011.1 software (Illumina Inc.). Quality parameters for DNA samples were the following: call rate >97, p10GC>0.7 (Guo et al., 2014). Quality parameters for SNVs were the following: call frequency 0.13-1.0, GenTrain 0.35-0.98, and ClusterSep higher than 0.27 (Illumina, 2010). Subsequent data analysis (Hardy-Weinberg equilibrium), SNV filtering, and SNV frequency calculations were performed using PLINK v1.9 software (Purcell et al., 2007).

Allele frequencies of effect variants included in our catalogue of effect variants were calculated and compared to the general European population (EUR) and distinct European populations (Utah residents with Northern and Western European ancestry [CEU]; Finnish in Finland [FIN]) based on the 1000 Genomes project data (1000 Genomes Project Consortium, 2015), which is accessible at the NCBI dbSNP database (Sherry et al. 1999). The general European population consisted of an aggregate of samples from all European populations, provided by the 1000 Genomes Project. This aggregate included genome data from CEU, FIN, British in England and Scotland, Iberian populations in Spanish, and Tuscany in Italy populations. Comparison with the general European population group was performed as the amount of differentiation within the European autosomal gene pool was found to be small (Lao et al., 2008). Particular CEU and FIN populations were chosen for the analysis according to Urnikyte et al. (2019), who showed that the Lithuanian population shares high proportions of ancestry components with the aforementioned populations. During the Urnikyte *et al*. (2019) study, a significant number of the candidate regions for positive selection detected in the Lithuanian population were also identified in FIN and/or CEU populations and thus pointed to common selection signals (Urnikyte *et al*., 2019). Allele frequencies of effect variants were compared using χ2 or Fisher’s exact test [when the sample size was ≤5], α=0.05, and Bonferroni multiple testing was performed. Statistical analysis was performed using Rstudio v3.5.2. software (R Core Team, 2013).

To define the possible impact on the genome, effect variants were analysed using *in silico* tools and databases: ClinVar (Landrum et al., 2018), Varsome (Kopanos et al., 2019), Uniprot (UniProt Consortium, 2019), Ensembl (Yates et al., 2020), and OMIM (https://omim.org/). Positive selection signature comparison with other populations was performed using the PopHumanScan database (Murga-Moreno et al. 2019), which contains data of positive selection signatures identified in many populations using different methods and the 1000 Genome Project phase 3 dataset.

The Ensembl database was used to compare pre-selected effect variants with primate species variants (*Gorilla gorilla, Pongo abelii, Theropithecus gelada,* and *Chlorocebus sabaeus*) to ascertain which allele was ancestral and which was derived (and potentially is more likely to be protective). To test if identified genome variants may be under recent positive selection and, therefore, may potentially be protective, we used RAiSD (Raised Accuracy in Sweep Detection), an open-source software that implements a novel and parameter-free detection mechanism that relies on multiple signatures of a selective sweep via the enumeration of SNV vectors. RAiSD calculates μ statistic, a test that combines three main distinct signatures that a sweep leaves in genomes - reduction of the polymorphism level, shift in the site frequency spectrum, and a localized pattern of linkage disequilibrium levels (Alachiotis and Pavlos, 2018), whereas other selection analysis methods are designed to detect one of the selection sweep signatures only. Another advantage of this tool is that it scans within the chosen cohort and does not require a reference population to detect a selective sweep. Finally, this tool does not demand high amounts of computational resources and offers parameter-free detection (Alachiotis and Pavlos, 2018). To infer or reject the potentially protective nature of identified genomic variants in our study, it was examined if identified genomic variants are in the regions of potential selective sweeps within the study population. All genotyping data of around 700,000 SNVs from the genotyping chips were used. If the μ values were above neutrality (μ=0), it was assumed, that the genomic region is under (high or low) recent positive selection. The higher the μ statistic value, the stronger the selective sweep signature is. The potency of selective sweep signals was evaluated qualitatively by comparing different signals throughout the chromosomes that were analysed. The assumption was made that the majority of the genome is neutral and the top 5% scores were chosen as candidate sweep regions. The top 5% corresponds to a p-value used as a cut-off threshold. All top 5% scores have p-values less than 0.05. This approach uses the empirical distribution of the scores and treats the majority of loci as control while the outlying 5% of the distribution is the candidate regions.

## Results

After quality control of the genotyping data, 465 samples were set for further analysis (10 samples did not reach the cut-off value of the call rate parameter). Filtered sequencing and genotyping data were also tested for the Hardy-Weinberg equilibrium. Out of 144 catalogue variants, only 70 were present in our dataset and met the filtering criteria (these variants were found in the Lithuanian population; 39 variants from genotyping and sequencing data, 7 variants from genotyping data alone, and 24 variants from sequencing data alone). The frequencies of four missense variants stood out as statistically significantly different between the study group and other European populations (Tables 1 and 2). Two other genome variants reached borderline significance in *PPARG* and *ADH1C* genes (p=0.05). After the Bonferroni multiple testing correction (p=2×10^-4^ 210 tests performed for 70 effect genome variants in three different population comparison groups [LTU vs CEU, LTU vs FIN, LTU vs EUR]) none of the variants reached statistical significance. This does not necessarily indicate that there are no significant associations. One of the major drawbacks of multiple-comparison studies is multiple testing. As a result, the significance of associations may be lost and potentially important data can be lost as well (Goldstein, 2009; Tam et al., 2019). This demonstrates the importance of targeted association analyses, where genomic regions of interest are specified and analyses are performed only for those specific regions. That is why we chose the aforementioned six suggestive-significant genome variants for further investigation.

**Table 1 -:** Effect variants and sample sizes. Sample sizes used for allele frequency analysis in the Lithuanian and European populations.

Variant ID	Gene	Related condition	LITGEN	CEU	FIN	EUR
rs1801282	*PPARG*	T2D	168	99	99	504
rs13266634	*SLC30A8*	T2D	464			
rs11556924	*ZC3HC1*	CHD	465			
rs2274223	*PLCE1*	Oesophageal cancer	463			
rs7498665	*SH2B1*	Obesity	98			
rs698	*ADH1C*	Alcohol dependence	98			

CEU - Utah residents (CEPH) with Northern and Western European ancestry; CHD - coronary heart disease, EUR - general European population, FIN - Finnish in Finland, LTU - Lithuanian population, T2D - type 2 diabetes.

**Table 2 -:** Comparison of frequencies of effect variants in the Lithuanian and European populations. Distribution of the effect allele genotypes and the statistics for the evaluation of differences in frequencies of effect variants in the Lithuanian and European populations. Only significant or borderline significant (*PPARG* and *ADH1C*) results are shown.

Variant ID	Gene	Change^*^	EP	HOMO ALT		HETERO		HOMO REF		MAF (LTU)	MAF (EP)	χ^2^	p
				LTU	EP	LTU	EP	LTU	EP				
rs1801282	*PPARG*	NM_001354668.2: c.34C>G	EUR	4	5	46	111	118	387	0,161	0,120	3,632	0,05
rs13266634	*SLC30A8*	NM_001172815.2: c.826C>T	CEU	50	6	195	36	219	57	0,318	0,242	4,387	0,04
rs11556924	*ZC3HC1*	NM_001282190.1: c.1025G>A	FIN	77	5	225	40	163	54	0,408	0,377	16,642	<0,01
rs2274223	*PLCE1*	NM_001165979.2: c.4856A>G	EUR	65	59	223	222	175	222	0,381	0,338	3,919	0,04
rs7498665	*SH2B1*	NM_001145812.1: c.1450A>G	EUR	19	52	36	227	43	224	0,378	0,329	7,015	0,03
rs698	*ADH1C*	NM_000669.5: c.1048A>G	EUR	25	93	48	221	25	189	0,5	0,405	5,878	0,05

EP - the population to which the comparison is made, CEU - Utah residents (CEPH) with Northern and Western European ancestry; EUR - general European population; FIN - Finnish in Finland; HETERO - heterozygous genotype count, HOMO ALT - homozygous alternative genotype count, HOMO REF - homozygous reference genotype count LTU - Lithuanian population; MAF - minor allele frequency.

^*^ All effect alleles were minor alleles.

According to the scientific literature, these missense variants may protect against alcohol dependence (*ADH1C*, rs698), type 2 diabetes (T2D) (*PPARG*, rs1801282; *SLC30A8*, rs13266634), coronary heart disease (CHD) (*ZC3HC1*, rs11556924), obesity (*SH2B1*, rs7498665), and oesophageal cancer (*PLCE1*, rs2274223).

**Figure 1 -:**
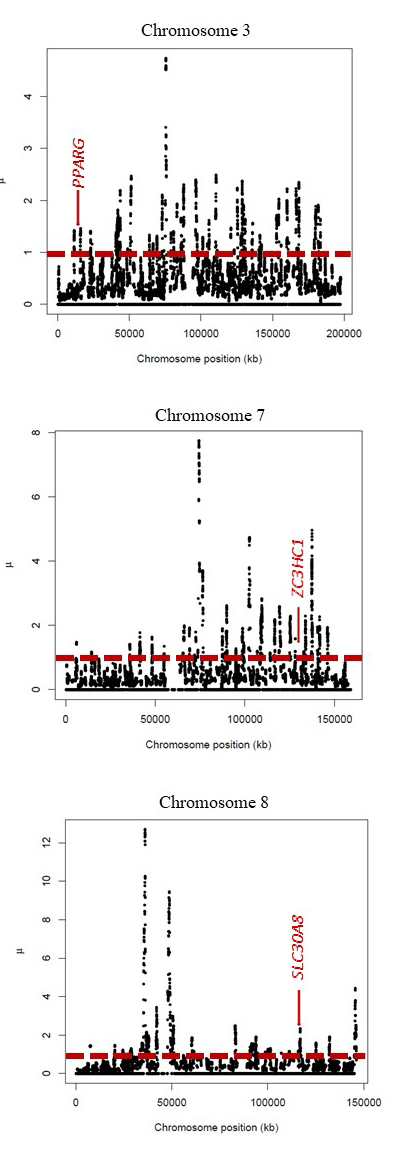
μ statistic curves for the chromosomes 3, 7, and 8. The highest μ statistic value point indicates the strongest recent positive selection (a selective sweep). Preliminary genomic locations of the candidate protective genes *PPARG* (chromosome 3), *ZC3HC1* (chromosome 7), and *SLC30A8* (chromosome 8) are indicated by the arrows. The dotted line indicates the cut-off value of the top 5% of μ values (μ=0.98 for chromosomes analysed).

Identified candidate effect variants were compared with primate species to ascertain which allele is derived and which is ancestral to avoid the erroneous assumption for common variants that the rare allele is the derived allele. The analysis showed that several of our catalogue-selected effect variants (in *PLCE1*, *ADH1C*, and *SH2B1* genes) in humans are ancestral, meaning their effect is more conservative. Finally, we have hypothesised that the derived alleles (in *PPARG*, *SLC30A8*, and *ZC3HC1* genes) tend to have more of a dynamic effect and may have a protective effect. To test this hypothesis, we have performed μ statistic test using RAiSD software (Alachiotis and Pavlos, 2018) to detect selective sweeps which might indicate recent positive selection in the aforementioned genes. Figure 1 shows the μ statistic curves for the chromosomes of *PPARG*, *SLC30A8,* and *ZC3HC1* genes. *SLC30A8* had a significant selective sweep signal (μ=2.34, p≥0.05). Genes *PPARG* and *ZC3HC1* did not reach the threshold of the top 5% values.

## Discussion

According to freely available *in silico* analysis tools, five of the six effect variants for which frequencies in the Lithuanian population differed from the European populations are considered benign (regarding Varsome or UniProt) or risk-determining (Ensembl). These five variants (*PPARG*: rs1801282, *SLC30A8*: rs13266634, *ZC3HC1*: rs11556924, *PLCE1*: rs2274223, *SH2B1*: rs7498665) were selected from scientific publications for our catalogue of the effect variants (Table S1). In these publications, variants were identified as potentially protective after GWAS data was filtered for nonsynonymous SNVs to increase the likelihood of them being functional. Besides, variants were considered protective when they were more frequent in the control group than in the study group. Evidence of positive natural selection for these variants was found and the probability of the variant being damaging was estimated (Butler et al., 2017). 

According to the Ensembl, ClinVar, and OMIM databases, the sixth variant (*ADH1C*: rs698) is classified as a protective variant with an impact on the metabolism of ethanol. However, studies suggest that this variant is associated with slower ethanol metabolism, which could lead to a longer period of consuming alcohol and the consumption of greater quantities. Therefore, people carrying the variant have a higher risk of heavy and excessive drinking (Edenberg, 2007; Tolstrup et al., 2008). In general, common SNVs could be responsible for as much as 30% of the variance in alcohol dependence, but only a few have been identified (Palmer et al., 2015). However, analyses indicate that additional SNVs associated with alcohol dependence are likely to have minor effects and are more consistent with more common psychiatric disorders (Walters et al., 2018). This shows that there is a lack of understanding of the molecular mechanisms involved in excessive alcohol consumption and other complex conditions and that the collection of large numbers of well-characterised cases and controls is needed. 

Besides function, the origin of the effect allele must also be addressed. A common practice to ascertain whether a nonsynonymous SNV is protective (i.e., the derived allele is protective) is to deduce which allele is derived and which is ancestral. A minor allele does not necessarily equal the derived (mutant) one, therefore the origin of the allele could be determined by using genomic alignments with primate species. The effect variants that were analysed were mostly not rare (MAF>0.5%). If a derived allele provides a protective function and gives an individual a selective advantage, one might expect positive selection to sweep it to become the most common allele in the population (Butler et al., 2017). This may be the reason why the effect variants that were analysed have allele frequencies greater than 0.5%. Moreover, this could explain why databases and SNV analysis tools call these variants as polymorphisms. Comparison with primate species showed that variants analysed in *PLCE1*, *ADH1C*, and *SH2B1* are indeed ancestral. The protective nature of genomic variants can be considered when the allele is derived, which is why we did not interpret these variants as protective and excluded these variants from further analysis. Despite contradicting data, significant variants still may have some effects on the etiopathogenesis of particular complex diseases.

Large-scale GWASs have identified a substantial number of genetic variants associated with T2D (Sanghera and Blackett, 2012), and only a few have been associated with protection against this disease. Our study indicates that the effect variant (rs13266634) in the *SLC30A8* gene may have an impact on protection against T2D. This gene encodes a protein that is involved in the optimisation of insulin secretion. Flannick et al. (2014) and Brunke-Reese et al. (2019) proposed that this variant, together with other less common, loss-of-function variants is associated with a lower-than-expected likelihood of T2D. Selective sweep signature analysis supports our hypothesis that this gene might have undergone recent natural selection. The μ statistic value for the *SLC30A8* gene reached the top 5% cut-off value of significance, although it is not as high as compared to other regions in chromosome 8. Nevertheless, the identification of weaker selective sweep signatures is not less important, as selective sweep has the potential to grow stronger and should be examined further. Also, the *SLC30A8* gene is not under positive selection in other populations, according to the PopHumanScan database (Murga-Moreno et al., 2019). This suggests that this selection signature may be important for the differentiation of the Lithuanian population.

A recent meta-analysis (Sarhangi et al., 2020) suggests that the variant rs1801282 in *PPARG*, which also emerged in our study, is associated with a decreased risk of T2D. SNVs of *PPARG* (nuclear receptor) have an important role in controlling lipid and glucose metabolism. The protective effect of the derived allele was detected to be significantly more common in some populations, including European (18%), East Asian (20%), and South-East Asian (18%) (Sarhangi *et al*., 2020). Moreover, our results correspond to this study, which suggests that Northern Europeans who carry the *PPARG* derived allele had a lower risk of T2D than Central or Southern Europeans. Moreover, T2D is closely related to CHD progression (Shadrina et al., 2020). During the frequency analysis step, we detected the *ZC3HC1* gene variant rs11556924, which is classified in scientific publications as most likely causing CHD. *ZC3HC1* is associated with the *KLHDC10* gene, which is involved in oxidative stress-induced cell death and inflammation. These processes are known to play a role in atherosclerosis and, in turn, CHD (Shadrina *et al*., 2020). However, an integrated haplotype score (Butler et al., 2017) for the effect variants in the *ZC3HC1* and *PPARG* genes showed that these variants may have undergone recent positive selection (Voight et al., 2006). This suggests that derived alleles could be beneficial for an individual’s fitness and may be protective. On the contrary, our study results of selective sweep analysis show no signature of recent positive selection for the *PPARG* and *ZC3HC1* gene loci (Figure 1).

*In silico* analysis tools and databases may describe effect variants not necessarily correctly and the data may not be up-to-date, inconsistent, and not relevant to all populations. This example of inconsistent findings on *ADH1C*, *PPARG,* and *ZC3HC1* and their variants’ effects shows the importance and need for the multi-level analysis approach for the effect variants. The study results of potential positive selection signatures may be strengthened and elaborated by performing the comparison of the results obtained with other populations (identified genomic loci under positive selection can be also under selective pressures in other populations) and/or different positive selection methods (e.g. Integrative Haplotype Score [iHS] or Cross Population Extended Haplotype Homozygosity [XP-EHH] analysis). A bigger dataset, i.e. whole-genome sequencing data, could also complement the analysis. It may give a better understanding of positive selection signatures in the Lithuanian population as it shows variation of the whole genome and not only specific genomic loci. To further investigate potential selection and account for demographic effects, simulations of demographic scenarios specific to the Lithuanian population would be required. This could be the aim of the follow-up study.

Additionally, it is important to keep in mind the effects of environmental factors. According to data from the Lithuanian Health Information Centre of the Institute of Hygiene (2021), diseases of the cardiovascular system caused the highest proportion of deaths (48.3%) in 2021 in Lithuania. In 2021, 5.57% of the population had T2D and 7.3% had CHD. In comparison, diseases of the cardiovascular system in the Finnish population were also one of the leading causes of death (33.9%) in 2019, according to the Statistics Finland. Estimates from a Finnish health survey in 2018 state that the prevalence of diabetes among adults over 30 years of age was 7.8% (Koponen et al., 2018). The majority of cases are of T2D (85%) (Sund and Koski, 2009). These diseases are more common among individuals whose reproductive period may be finished meaning an increase in lethality of these individuals should not affect the fitness of the individuals in the next generation too much. However, some individuals have these diseases at a young age (Pulgaron and Delamater, 2014; De Venecia et al., 2016). In this case, the next generations may benefit from the knowledge of positive selection signatures in the loci related to these diseases. Also, some of the younger individuals who do not have these conditions although living a high-risk lifestyle, may hold disease-specific protective genome variation, and specific loci under positive selection may protect from a disease from generation to generation in a population. Even though our population gene pool holds effect variants that may protect against these complex diseases, lifestyle, and other environmental factors influence the frequency of morbidity. This contradiction of the high frequency of described diseases and present protective genomic variation could also suggest that the ancestors of the Lithuanian population faced unique selective influences, especially in relation to genes associated with energy metabolism. Furthermore, analysed genes may influence on other diseases and phenotypes as well. For instance, *ZC3HC1* is associated with rheumatoid arthritis (López-Mejías et al., 2013) while *PPARG* is associated with various types of cancer (Ogino et al., 2009; Ahmad et al., 2016; Goldstein et al., 2017). However, these genes are most commonly associated with the diseases which were discussed in this article. 

## Conclusions

We identified a plausible effect variant rs13266634 in *SLC30A8* in the Lithuanian population group that may protect against T2D. In addition, we suggested a new analysis strategy for the evaluation of genome variants. A better understanding of common variation and its effects can help build more informative databases and avoid sometimes misleading information on the effects of the variant, as demonstrated by this study with the *ADH1C*, *PPARG*, and *ZC3HC1* gene variants. Identification of effect variants is crucial for a better understanding of etiopathogenetic mechanisms and microevolutionary processes. Many studies analyse coding effect variants and important interactions between coding and non-coding variants remain understudied (Kido et al., 2018). Thus, when we define the underlying genetic population structure, we should further move our research toward the intricate genetic mechanisms, processes, and interactions that control the balance between health and disease. Analysis of effect variants can broaden knowledge about the differences between populations and tackle some problems regarding personalised medicine. Population-specific effect variants can become targets for the development of disease prevention programs and novel therapies and the use of genome editing tools.

## Supplementary material

The following online material is available for this article:

Table S1 - Catalogue of effect variants.
