# Supplementary material for: Putative protective genomic variation in the Lithuanian population
Source: Genet Mol Biol. 2024 Apr 15;47(2):e20230030. doi: 10.1590/1678-4685-GMB-2023-0030 (PMC11021042; doi:10.1590/1678-4685-GMB-2023-0030)
Supplement: Table S1 - [file 1415-4757-GMB-47-02-e20230030-s1.pdf]

Genetics and Molecular Biology
https://doi.org/10.1590/1678-4685-GMB-2023-0030

Supplementary Material to "Putative protective genomic variation in the Lithuanian population"

Table S1 - Catalogue of effect variants.

Block*	Gene	rs (dbSNP)	Reference allele	Alternative allele	Associated disease or trait
1	*IL7R*	rs6897932	C	T	Multiple sclerosis
	COL11A1	rs3753841	G	A	Glaucoma (primary open-angle)
	SLC30A8	rs13266634	C	T	Type 2 diabetes
	TYK2	rs12720356	A	C	Psoriasis
	KCNE4	rs12621643	T	G	Acute lymphoblastic leukemia (childhood)
	IL17REL	rs5771069	G	A	Ulcerative colitis
	PLCE1	rs3765524	T	C	Esophageal cancer and gastric cancer
	TNFSF13	rs3803800	A	G	IgA nephropathy
	WDFY4	rs7097397	G	A	Systemic lupus erythematosus
	PLCE1	rs2274223	G	A	Esophageal cancer
	CFB	rs641153	G	A	Age-related macular degeneration
	CD6	rs11230563	C	T	Inflammatory bowel disease
	KLRC4	rs2617170	T	C	Behcet's disease
	ZC3HC1	rs11556924	C	T	Coronary heart disease
	TYK2	rs2304256	C	A	Type 1 diabetes
	GIPR	rs1800437	G	C	Obesity
	C2	rs9332739	G	C	Age-related macular degeneration
	PPARG	rs1801282	C	G	Type 2 diabetes
	TUBD1	rs1292053	G	A	Inflammatory bowel disease
	MMP17	rs6598163	G	A	Migraine
	LOXL1	rs3825942	G	A	Glaucoma (exfoliation)
	IL23R	rs11209026	G	A	Inflammatory bowel disease
	MPV17L2	rs874628	A	G	Multiple sclerosis
	RTKN2	rs3125734	T	C	Rheumatoid arthritis
	LRRC34	rs6793295	C	T	Interstitial lung disease
	SH2B1	rs7498665	G	A	Obesity
	ANKLE1	rs8100241	G	A	Breast cancer
	BANK1	rs10516487	G	A	Systemic lupus erythematosus

Block*	Gene	rs (dbSNP)	Reference allele	Alternative allele	Associated disease or trait
	ANKLE1	rs2363956	T	G	Ovarian cancer
	NFKBIE	rs2233434	G	A	Rheumatoid arthritis
	LPL	rs328	C	G	Hypertension
	WFS1	rs1801214	T	C	Type 2 diabetes
	THADA	rs7578597	T	C	Type 2 diabetes
	IL13	rs20541	G	A	Psoriasis
	MLPH	rs2292884	G	A	Prostate cancer
2	*PCSK9*	rs11591147	G	T	Hypercholesterolemia
	PCSK9	rs67608943	C	G	Hypercholesterolemia
	PCSK9	rs72646508	C	T	Hypercholesterolemia
	PCSK9	rs28362263	G	A	Hypercholesterolemia
	SCN9A	rs202152511	C	T	Congenital pain disorders:for example, inherited erythromelalgia
	SCN11A	rs483352920	T	C	Congenital pain disorders:for example, inherited erythromelalgia
	DYNC2LI1	rs769975073	G	A	Sclerosteosis
	DYNC2LI1	rs201948500	C	G	Sclerosteosis
	LPA	rs41272114	G	A	Cardiovascular disease
	LPA	rs10455872	T	C	Cardiovascular disease
	LPA	rs3798220	T	C	Cardiovascular disease
	TYK2	rs34536443	C	G	Reduced risk of rheumatoid arthritis, systemic lupus erythematosus
	TYK2	rs35018800	C	T	Reduced risk of rheumatoid arthritis, systemic lupus erythematosus
	TYK2	rs12720356	T	G	Reduced risk of rheumatoid arthritis, systemic lupus erythematosus
3	*ACKR1*	rs2814778	T	C	White blood cell count quantitative trait locus, resistance to plasmodium vivax
	ADH1B	rs2066702	C	T	Alcohol dependence
	ADH1B	rs1229984	T	C	Alcohol dependence, aerodigestive tract cancer, squamous cell
	ADH1C	rs698	A	G	Alcohol dependence
	ADH1C	rs1693482	G	A	Alcohol dependence
	ALDH2	rs671	G	A	Alcohol dependence, acute alcohol sensitivity, susceptibility to hangover, susceptibility to poor response to sublingual

Block*	Gene	rs (dbSNP)	Reference allele	Alternative allele	Associated disease or trait
					nitroglycerin, esophageal cancer
	APOC3	rs140621530	G	T	Hyperalphalipoproteinemia 2, coronary heart disease
	APOC3	rs147210663	G	A	Hyperalphalipoproteinemia 2, coronary heart disease
	APOC3	rs138326449	G	A	Hyperalphalipoproteinemia 2, coronary heart disease
	APOC3	rs76353203	C	T	Hyperalphalipoproteinemia 2, coronary heart disease
	APP	rs63750847	G	A	protection against Alzheimer disease
	C2	rs547154	G	T	Age-related macular degeneration
	CASP8	rs3834129	AGTAAG	-	protection against lung cancer
	CCR2	rs1799864	G	A	Susceptibility to HIV type 1
	CCR5|LOC 102724297	rs1800940	G	T	Susceptibility to HIV type 1
	CCR5|LOC 102724297	rs1799987	A	G	Susceptibility to HIV type 1
	CCR5|LOC 102724297	rs1800560	T	A	Susceptibility to HIV type 1
	CCR5|LOC 102724297	rs333	ACAGTC AGTATCA ATTCTGG AAGAAT TTCCAGA	-	Susceptibility to HIV type 1, West nile virus, resistance to hepatitis C virus, multiple sclerosis modifier of disease progression
	CX3CR1	rs3732378	G	A	HIV type 1, rapid progression to AIDS, resistance to coronary artery disease, susceptibility to macular degeneration
	CX3CR1	rs3732379	C	T	HIV type 1, rapid progression to AIDS, resistance to coronary artery disease, susceptibility to macular degeneration
	CXCL12	rs387906400	G	A	Susceptibility to HIV type 1

Block*	Gene	rs (dbSNP)	Reference allele	Alternative allele	Associated disease or trait
	FCGR2B	rs1050501	T	C	Susceptibility to systemic lupus erythematosus, resistance to malaria
	GABBR2	rs1435252	G	A	Protection against nicotine dependence
	GABBR2	rs3780422	C	T	Protection against nicotine dependence
	GABBR2	rs2779562	T	C	Protection against nicotine dependence
	GABBR2	rs3750344	T	C	Protection against nicotine dependence
	HBB	rs334	A	T	Beta-thalassemia, Heinz body anemia, Hb SS disease, malaria
	HBB	rs33950507	G	A	Hb SS disease, resistance to malaria, beta thalassemia disease
	HBB	rs33930165	G	A	Hb SS disease, resistance to malaria
	IGF1R	rs33958176	G	A	Resistance to insulin-like growth factor 1
	IGF1R	rs121912429	G	A	Resistance to insulin-like growth factor 1
	IGF1R	rs121912428	C	T	Resistance to insulin-like growth factor 1
	IGF1R	rs121912427	A	C	Resistance to insulin-like growth factor 1
	IGF1R	rs121912426	G	A	Resistance to insulin-like growth factor 1
	IL4R	rs1805015	T	C	Resistance to atopy
	IL4R	rs1805010	A	G	Resistance to atopy, slow progression to acquired immunodeficiency syndrome
	KCNMB1| KCNIP1	rs11739136	G	A	Resistance to hypertension
	MC4R	rs52820871	A	C	Obesity, monogenic diabetes
	MT-ND3	rs2853826	A	G	Resistance to Parkinson's disease
	ODC1	rs2302615	G	A	Reduced risk of colonic adenoma recurrence
	PKHD1	rs137852944	C	T	Protection against autosomal recessive polycystic kidney disease, colorectal cancer, polycystic kidney dysplasia, oligohydramnios, periportal fibrosis
	PRNP	rs267606980	G	T	Protection against Kuru

Block*	Gene	rs (dbSNP)	Reference allele	Alternative allele	Associated disease or trait
	PRSS2|TRB	rs61734659	G	A	Protection against pancreatitis
	SLC30A8	rs587777582	AAGATCA	-	Diabetes mellitus type 2
	SLC30A8	rs200185429	C	T	Diabetes mellitus type 2
	SLC4A1	rs769664228	CGGCAGCCAGGACCTGGGGGCTGAATGC	-	Resistance to Malaria; Stomatocytic elliptocytosis
	SSTR5	rs121917877	C	T	Resistance to somatostatin analog
	TIRAP	rs8177374	C	T	Protection against Invasive pneumococcal disease, resistance to malaria, protection against Mycobacterium tuberculosis, resistance to bacteremia
	TLR1	rs5743618	G	T	Protection against leprosy
	TLR5	rs5744168	C	T	Resistance to legionellosis, systemic lupus erythematosus, melioidosis
	TOP2A	rs267607133	G	A	Dna topoisomerase II, resistance to inhibition of, by amsacrine
	BDNF|BDNF-AS	rs6265	G	A	Protection against obsessive-compulsive disorder
	FUT2|LOC105447645	rs601338	G	A	Resistance to Norwalk virus infection
	TLR3	rs3775291	C	T	Susceptibility to HIV type 1
	TOR1A	rs1801968	G	C	Dystonia 1, modifier of torsion
	NOD2	rs104895421	T	A	Blau syndrome, Chrohn's disease, sarcoidosis
	NOD2	rs104895422	C	T	Blau syndrome, Chrohn's disease, sarcoidosis
	NOD2	rs104895427	C	T	Blau syndrome, Chrohn's disease, sarcoidosis
	NOD2	rs104895430	G	A	Blau syndrome, Chrohn's disease, sarcoidosis
	NOD2	rs104895456	G	A	Blau syndrome, Chrohn's disease, sarcoidosis
	NOD2	rs104895467	A	G	Blau syndrome, Chrohn's disease, sarcoidosis
	NOD2	rs104895486	C	T	Blau syndrome, Chrohn's disease, sarcoidosis

Block*	Gene	rs (dbSNP)	Reference allele	Alternative allele	Associated disease or trait
	CFH	rs1061170	C	T	Membranoproliferative glomerulonephritis with complement factor h deficiency, atypical hemolytic-uremic syndrome 1, basal laminar drusen, Age-related macular degeneration 4
	IL2RA	rs11594656	T	A	Diabetes mellitus, insulin-dependent, 10
	EGFR	rs121434569	C	T	Lung cancer, drug response
	IGF1R	rs1253103806	G	A	Resistance to insulin-like growth factor 1
	UMOD	rs13333226	A	G	Hypertension, diabetes
	IGF1R	rs1409058783	G	T	Resistance to insulin-like growth factor 1
	IRF5	rs1432329681	T	-	Susceptibility to systemic lupus erythematosus, rheumatoid arthritis
	PRSS1	rs143909348	G	A	Hereditary pancreatitis
	PCSK9	rs1553137557	GC	AA	Familial hypercholesterolemias
	GYPC	rs1553470034	CAGAGCCTGATCCAGGGATGTCTGGATGGCCGGATGGCAGAATGGAGACCTCCACCCCCACCATAATGGACATTGTCGTCATTG	-	Resistance to malaria
	IGF1R	rs1555434208	G	A	Resistance to insulin-like growth factor 1
	IGF1R	rs1555460945	G	T	Resistance to insulin-like growth factor 1
	SLC45A2	rs16891982	C	G or C	Melanoma, basal cell carcinoma, sensitivity to sun
	COL1A1	rs1800012	C	A	Bone mineral density variation
	NQO1	rs1800566	G	A	Alcohol consumption, cancer
	IL10	rs1800872	T	G	Resistance to graft-versus-host disease, susceptibility to HIV type 1

Block*	Gene	rs (dbSNP)	Reference allele	Alternative allele	Associated disease or trait
	FCGR2A	rs1801274	A	T or G	Ankylosing spondylitis, Chrohn disease, Inflammatory bowel disease, systemic lupus erythematosus, Kawasaki disease, Lupus nephritis, Ulcerative colitis
	TG	rs180195	A	G	Autoimmune thyroid disease
	IRS2	rs1805097	C	T	Diabetes mellitus type 2
	NOS3	rs2070744	C	T	Susceptibility to coronary artery spasm, metabolic syndrome
	MC4R	rs2229616	C	T	Diabetes, obesity
	CLEC1A	rs2306894	G	C	Aspergillosis
	TAB2	rs237025	G	A	Diabetes mellitus, insulin-dependent, 5
	UGT1A1	rs3064744	TA	TA[9]	Crigler-Najjar syndrome, type II, Lucey-Driscoll syndrome, Gilbert's syndrome, not provided, not specified, Irinotecan response, Bilirubin, serum level of, quantitative trait locus 1
	CTLA4	rs3087243	G	A	Hashimoto thyroiditis, Celiac disease
	HBB	rs33945705	G	A or T	Hemaglobin structure changes
	HBB	rs33946267	C	T or G or A	Beta-thalassemia, Hb SS disease
	TLR1	rs4833095	T	C	Asthma and hay fever, leprosy
	SCN8A	rs587780455	A	G	Seizures, epilepsy
	LAMA3	rs61751706	C	T	Junctional epidermolysis bullosa gravis of Herlitz, Laryngo-onycho-cutaneous syndrome
	FGG	rs75848804	C	G	Fibrinogen Osaka V
	LDLR	rs771917370	C	T or G	Hypercholesterolemia
	SOD1	rs80265967	A	C	Amyotrophic lateral sclerosis

* Effect variants were selected from different scientific sources.
Block number 1 was selected from the article by Butler JM et al. Identification of candidate protective variants for common diseases and evaluation of their protective potential. BMC Genomics. 2017; 18(1):575.
Block number 2 was selected from the article by Harper AR et al. Protective alleles and modifier variants in human health and disease. Nature Reviews Genetics. 2015; 16(12):689–701.BMC Genomics. 2017; 18(1):575.

Block number 3 was selected from the ClinVar database.
